# A Rare Case of Recurrent Intestinal Obstruction in a Young Female: Abdominal Cocoon

**DOI:** 10.7759/cureus.105245

**Published:** 2026-03-14

**Authors:** Saba Rupani, Saroj K Sahoo, Shambhavi Prakash, Khaja Abdul Malik Adeel, Vedaant Parekh

**Affiliations:** 1 General Surgery, Kalinga Institute of Medical Sciences, Bhubaneswar, IND; 2 General Surgery, Dr. Shankarrao Chavan Government Medical College and Hospital, Nanded, IND

**Keywords:** abdominal cocoon syndrome, case report, fibrous membrane, laparoscopy, recurrent intestinal obstruction, sclerosing encapsulating peritonitis

## Abstract

Abdominal cocoon syndrome (ACS), also known as sclerosing encapsulating peritonitis (SEP), is an uncommon cause of intestinal obstruction characterized by partial or total encapsulation of the small intestine by a fibrous membrane. It typically presents with recurrent or subacute episodes of bowel obstruction and is often diagnosed intraoperatively due to the non-specific nature of clinical and radiologic findings. A 17-year-old girl presented with symptoms of acute intestinal obstruction, including abdominal pain, vomiting, and obstipation. She had experienced similar episodes over the past year. Initial investigations were inconclusive, and though emergency laparotomy was considered, her symptoms transiently improved. Thus, an elective diagnostic laparoscopy was later performed, revealing the presence of an encapsulating fibrous membrane consistent with ACS. The membrane was dissected and removed. Histopathology showed fibrocollagenous tissue with inflammation but no granuloma or malignancy. The patient recovered well postoperatively, with a one-month follow-up uneventful. ACS remains a diagnostic challenge due to its rarity and non-specific presentation. Laparoscopic intervention serves as both a diagnostic and therapeutic tool, particularly in young patients with recurrent, unexplained episodes of obstruction.

## Introduction

Abdominal cocoon syndrome (ACS), also known as sclerosing encapsulating peritonitis (SEP), is a rare but clinically significant cause of recurrent or subacute intestinal obstruction. First described by Foo et al. in 1978, the condition is characterized by partial or complete encasement of the small intestine by a dense fibrocollagenous membrane, resulting in impaired bowel motility and mechanical obstruction [1]. Although cases have been reported across various age groups, idiopathic abdominal cocoon predominantly affects adolescent girls and young adults, particularly in tropical and subtropical regions [2-5].

The etiology is broadly classified into idiopathic and secondary forms. While the pathogenesis of idiopathic SEP remains unclear, proposed mechanisms include retrograde menstruation, subclinical peritonitis, viral infections, and autoimmune peritoneal reactions [1,6]. Secondary SEP may arise due to peritoneal dialysis, previous abdominal surgeries, tuberculosis, ventriculoperitoneal shunts, chronic β-blocker use, or systemic inflammatory diseases [3,6-8]. The broad spectrum of etiologies contributes to the diagnostic difficulty, as the condition often mimics other intra-abdominal pathologies.

Clinically, abdominal cocoon presents with recurrent abdominal pain, vomiting, distension, and features of intermittent or chronic intestinal obstruction [2,3,6]. These symptoms are non-specific and overlap with conditions such as adhesive small bowel obstruction, Crohn's disease, internal hernia, tuberculosis-related strictures, peritoneal carcinomatosis, and encapsulating peritoneal sclerosis [6-8]. Physical examination is often unremarkable, further complicating early recognition.

Radiologic evaluation plays an important supportive role. Contrast-enhanced computed tomography (CECT) is considered the most useful modality, with hallmark findings including clustering of small bowel loops, a thick-enhancing fibrous membrane, peritoneal thickening, loculated ascites, or fixation of loops in a concertina-like fashion [6,9,10]. However, these features may be subtle and are frequently overlooked, resulting in preoperative diagnostic accuracy remaining low [3,6,11].

Management depends on severity, but surgical intervention remains the definitive treatment in symptomatic patients. Laparotomy or laparoscopy typically reveals the characteristic encasing membrane, and treatment involves careful adhesiolysis and complete excision of the fibrous sac [3,6,12,13]. Complications may include iatrogenic enterotomy, postoperative ileus, or short-term recurrence; however, prognosis after complete membrane removal is generally excellent [2,3,6].

## Case presentation

A 17-year-old girl presented to our emergency department with complaints of right lower abdominal pain, vomiting, and obstipation for the past 24 hours. She reported similar episodes of abdominal pain and vomiting over the past year, none of which led to a definitive diagnosis. Her past surgical history included a laparoscopic appendectomy performed nine months earlier in November.

On examination, the patient appeared moderately ill but was hemodynamically stable. Abdominal examination revealed diffuse tenderness and mild central distension without signs of peritonitis. The patient reported similar episodes of abdominal pain and obstruction occurring one week, six months, and nine months prior to presentation, all of which resolved with conservative treatment but without a definitive diagnosis. Ultrasonography of the abdomen was non-contributory. Routine laboratory investigations, including complete blood count, renal and liver function tests, and serum electrolytes, were within normal limits.

**Figure 1:**
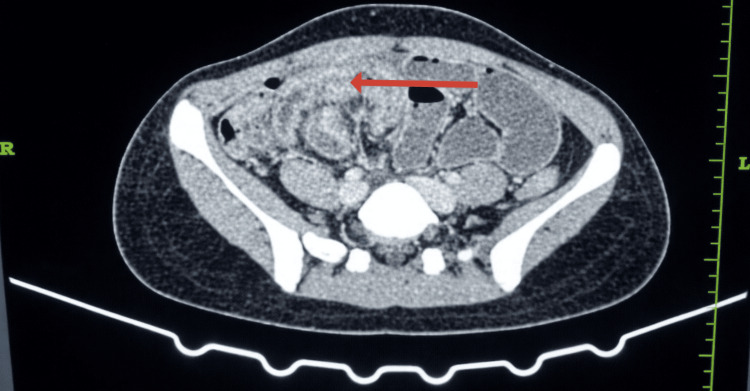
Enhancing membrane (cocoon) on CECT The dilated proximal bowel loops filled with gas are marked with an arrow. CECT: contrast-enhanced computed tomography

**Figure 2:**
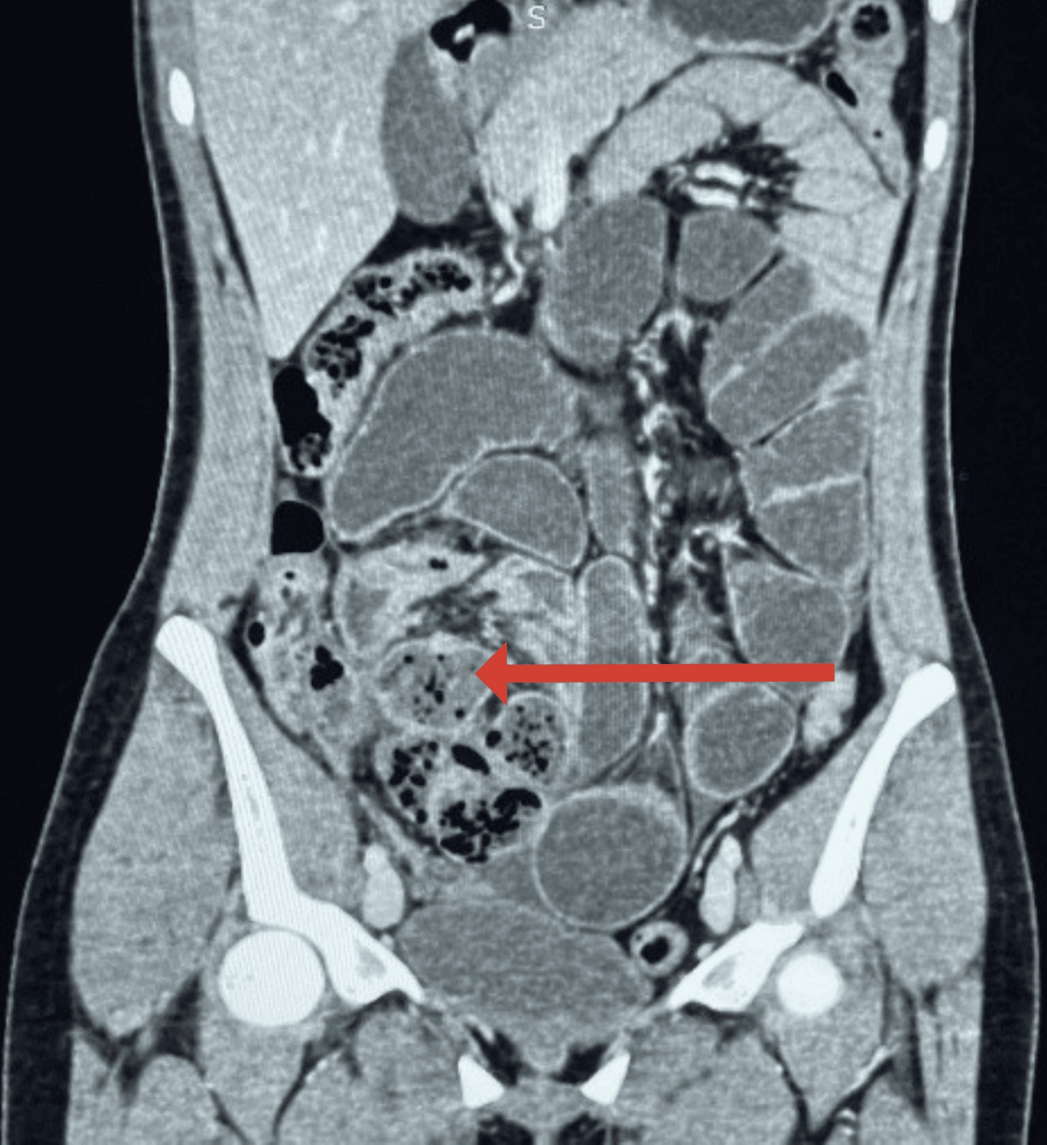
Coronal section on CECT showing similar findings, as marked by an arrow CECT: contrast-enhanced computed tomography

**Figure 3:**
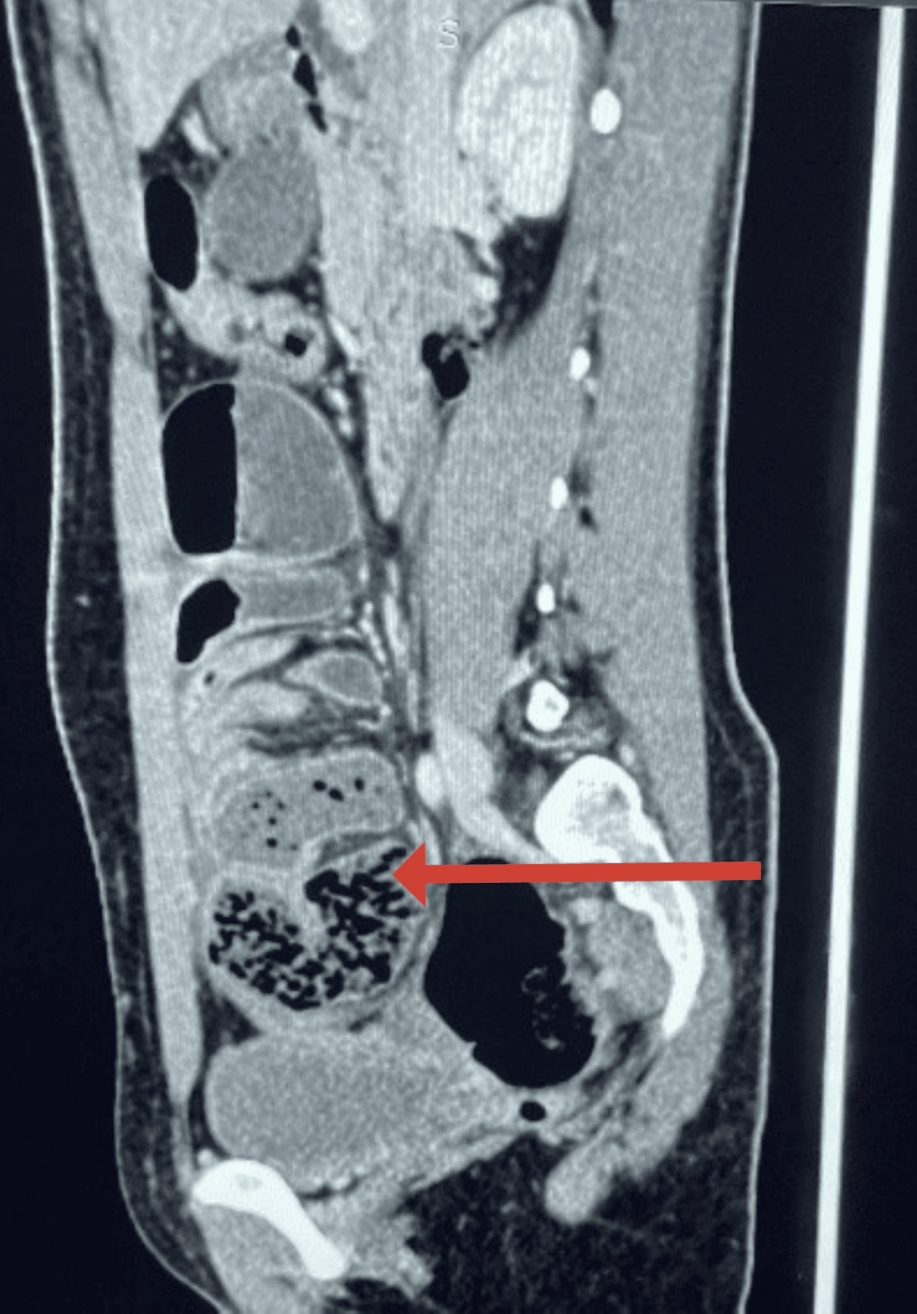
Saggital section on CECT Sagittal CECT image showing encapsulated bowel loops in close proximity to the urinary bladder and uterus, emphasizing the potential surgical implications due to involvement of adjacent pelvic structures (as seen by the arrow). CECT: contrast-enhanced computed-tomography

CECT of the abdomen and pelvis showed dilated proximal bowel loops filled with gas. The distal ileal loops were clustered and encapsulated by a surrounding enhancing membrane, predominantly localized in the right iliac fossa. These findings raised strong concern for ACS. A representative axial CT image showing these features is correlated with intraoperative findings (Figure 1). However, there was no definitive transition point or features suggestive of malignancy or inflammatory bowel disease. Similar findings were confirmed on the coronal section (Figure 2). The sagittal section of the CECT scan holds particular surgical relevance. It demonstrates that the encapsulated bowel loops are abutting the urinary bladder and, to some extent, the uterus (Figure 3). This close anatomical relationship is crucial to consider during laparoscopic dissection and membrane excision to avoid inadvertent injury to adjacent pelvic structures. The patient had an uneventful postoperative recovery and was discharged on postoperative day 10. Histopathological examination of the excised membrane revealed fibrocollagenous tissue with areas of hyalinization, necrosis, and inflammatory cell infiltration. No granuloma or malignancy was identified. At one-month follow-up, the patient was asymptomatic and had resumed a normal diet and activities.

Although emergency laparotomy was initially considered on day one, clinical improvement was observed over 24 hours with conservative management including intravenous fluids and nasogastric decompression. Elective surgical intervention was thus planned on day three, and an elective diagnostic plus therapeutic three-port laparoscopy was performed.

**Figure 4:**
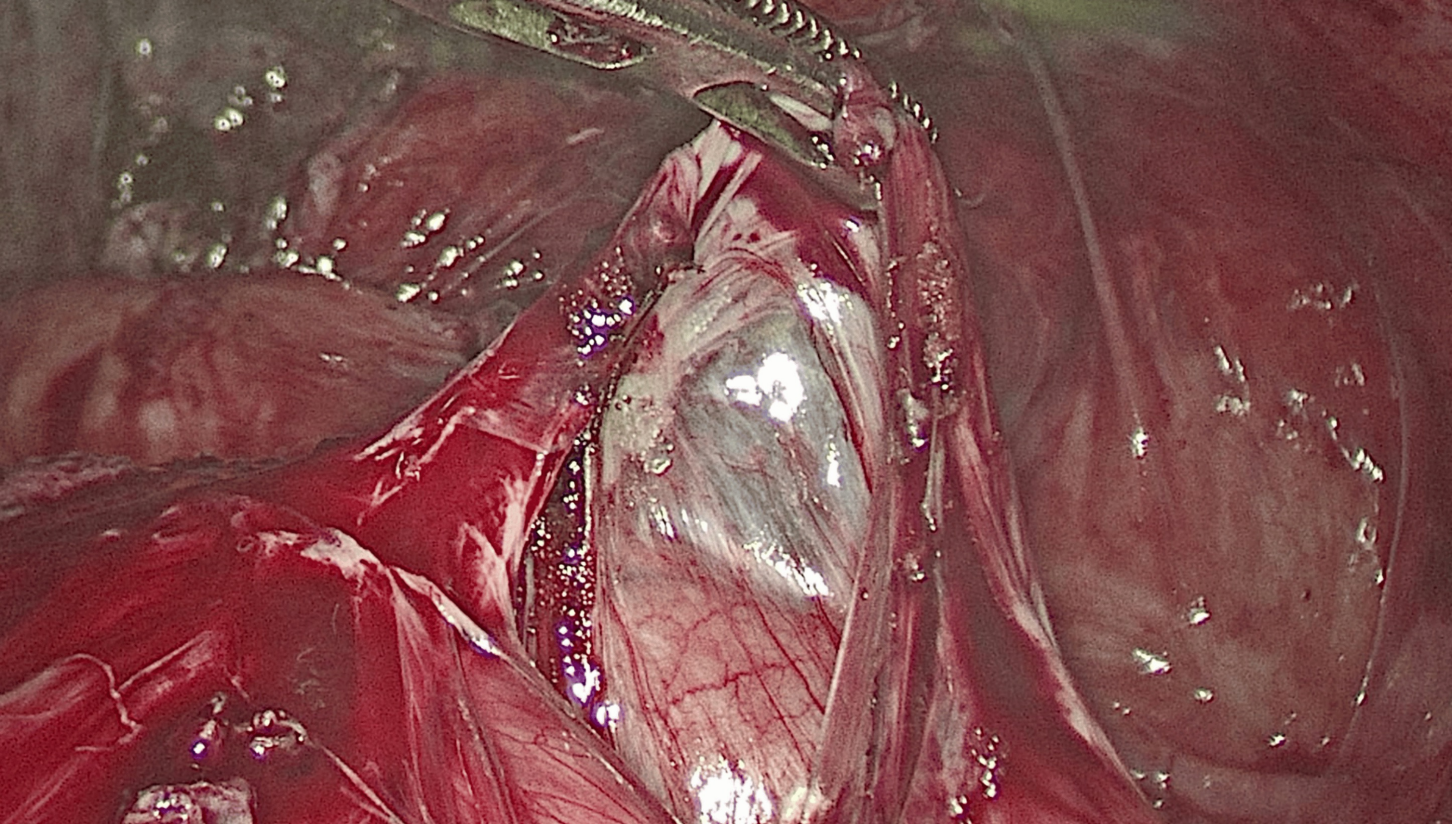
Abdominal cocoon Intra-operative image showing abdominal cocoon characterized by a thick fibrocollagenous membrane encapsulating the small bowel loops, which is being dissected by meticulous adhesiolysis, held by a bowel grasper, as shown in the image.

Diagnostic laparoscopy was initiated and revealed no additional visceral abnormalities. Intraoperatively, the small bowel was found to be encased in a dense, whitish, fibrocollagenous membrane, forming a sac-like structure, as seen in Figure 4, the classic hallmark of ACS. The membrane appeared tense, chitinous, and fibrotic, with no surrounding fluid collection, favoring a primary (idiopathic) variant.

Dissection was initiated in the right iliac fossa by mobilizing the cecum, and adhesiolysis was carried out in a cranial direction toward the mid-abdomen. Meticulous and steady adhesiolysis was performed to free the encapsulated ileal loops. As dissection in this plane can be technically demanding, the surgical team adopted a cautious approach, avoiding overly fibrotic or unclear planes and reattempting entry from safer adjacent sites when necessary.

Eventually, the correct anatomical plane was accessed, and the fibrotic membrane was dissected completely. The entire procedure lasted approximately three hours. All parts of the cocoon were separated meticulously up to the ileocecal junction and were removed in toto. There was no evidence of bowel necrosis or enterotomy, and the procedure was successfully completed laparoscopically.

The excised specimen was sent for histopathological examination. The peritoneal cavity was irrigated thoroughly, and a final survey of the pelvis revealed no other visceral pathology.

**Figure 5:**
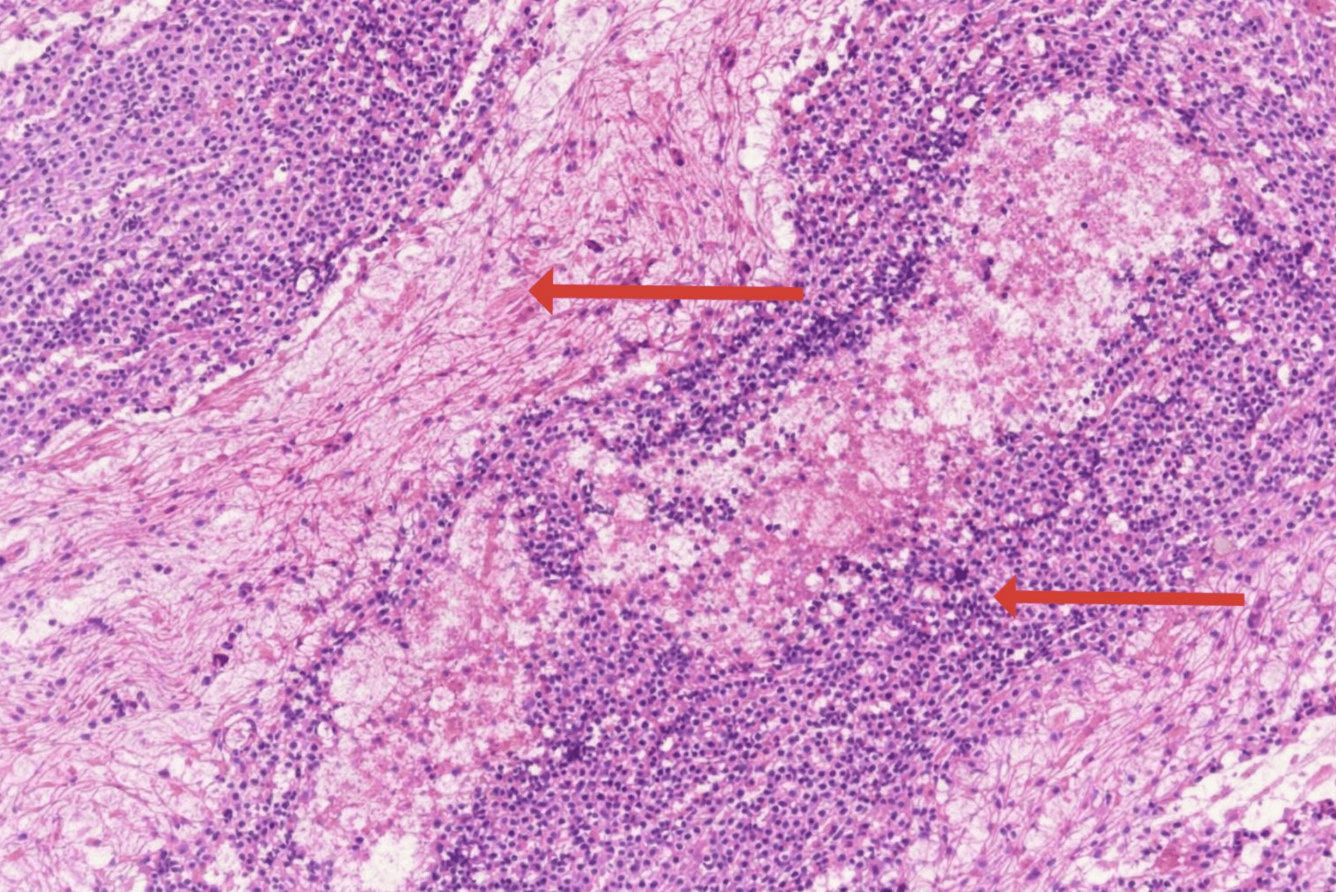
Histopathological examination of the excised membrane (H&E stain) Photomicrograph showing pink thread-like fibrocollagenous tissue (left arrow) with areas of hyalinization. Blue-stained inflammatory cells are seen infiltrating the tissue (right arrow), with focal areas of necrosis. No granuloma or malignant cells are identified (Hematoxylin and eosin stain, original magnification ×10).

The patient had an uneventful postoperative recovery and was discharged on postoperative day seven. Histopathological examination of the excised membrane revealed fibrocollagenous tissue with areas of hyalinization, necrosis, and inflammatory cell infiltration, as seen in Figure 5. No granuloma or malignancy was identified. At one-month follow-up, the patient was asymptomatic and had resumed a normal diet and activities.

## Discussion

ACS is a rare but significant cause of intestinal obstruction, especially in younger females in tropical and subtropical regions. It has been classified into three types based on the extent of peritoneal encapsulation. In type I, only a segment of the small intestine is enclosed. In type II, the entire small intestine is encapsulated. In type III, the membrane extends beyond the small bowel to include other abdominal organs such as the stomach, appendix, cecum, ascending colon, or ovaries [6]. The exact cause of idiopathic abdominal cocoon remains unknown. The proposed mechanisms include retrograde menstruation, subclinical peritonitis, or autoimmune processes [1]. The secondary causes may involve peritoneal dialysis, previous abdominal surgeries, tuberculosis, or the use of certain medications [7,8].

In most cases, the diagnosis is made intraoperatively, although imaging modalities, such as CECT, may raise suspicion by showing clustering of small bowel loops within a membrane or “cocoon” and displacement of surrounding structures [6,9]. However, such findings are often subtle, as seen in this case, where imaging showed an enhancing membrane but lacked definitive features.

The gold standard of treatment involves surgical removal of the fibrous membrane and release of the bowel. While traditional management involved open surgery, recent literature suggests that laparoscopy is both diagnostic and therapeutic, offering the benefits of less postoperative pain, shorter hospital stay, and better cosmetic results [12,14]. In our case, the laparoscopic approach proved successful in confirming the diagnosis, excising the membrane, and ensuring complete resolution.

Histopathology typically shows dense fibrocollagenous tissue with chronic inflammatory infiltrate, occasionally with evidence of granuloma in secondary forms (e.g., tuberculosis). In our case, the absence of granuloma or malignancy was consistent with an idiopathic variant [6,7,9].

ACS is an uncommon clinical entity that continues to be reported mainly through isolated case reports and small case series. Since its initial description by Foo et al. in 1978, awareness of this condition has gradually increased, though it remains underdiagnosed due to its nonspecific clinical presentation [1]. Most reported cases involve young females presenting with recurrent or subacute intestinal obstruction, often without clear predisposing factors, which closely mirrors the presentation in the current case [2,3].

The idiopathic form of ACS is more frequently encountered in adolescents and young adults, particularly in tropical regions, while secondary forms are typically associated with identifiable etiologies, such as peritoneal dialysis, tuberculosis, prior abdominal surgery, ventriculoperitoneal shunts, and chronic inflammatory conditions [2,6,7]. Although the exact pathogenesis of idiopathic ACS remains uncertain, the proposed mechanisms include subclinical peritonitis, autoimmune-mediated inflammation, and retrograde menstruation, leading to a fibrotic peritoneal response [1,6,9].

Radiological diagnosis remains challenging. While CECT is considered the most informative imaging modality, its findings may be subtle and easily overlooked. Characteristic CT features described in the literature include clustering and fixation of small bowel loops, encasement by a fibrous membrane, peritoneal thickening, loculated ascites, and displacement of surrounding abdominal organs [6,9,15]. Despite these findings, many authors emphasize that a high index of clinical suspicion is essential, as preoperative diagnosis is frequently missed [3,6].

Surgical management remains the definitive treatment for symptomatic ACS. Complete excision of the fibrous membrane with meticulous adhesiolysis is associated with excellent outcomes and low recurrence rates [3,6]. Routine bowel resection is discouraged unless there is clear evidence of ischemia or nonviability, as unnecessary resection may increase postoperative morbidity [6,7]. In recent years, laparoscopy has gained acceptance as both a diagnostic and therapeutic modality in selected patients, offering advantages such as reduced postoperative pain, shorter hospital stay, and faster recovery [12,14].

Histopathological findings across reported cases are largely consistent, typically demonstrating dense fibrocollagenous tissue with chronic inflammatory infiltrates and varying degrees of hyalinization. Granulomatous inflammation is more suggestive of secondary causes, particularly tuberculosis, whereas idiopathic cases lack granulomas or malignant features [6,7,9]. Overall prognosis after complete surgical excision is favorable, with most patients remaining asymptomatic on long-term follow-up [2,3,6].

## Conclusions

This case highlights ACS as a rare but important differential diagnosis in young patients presenting with recurrent, unexplained abdominal pain and features of intestinal obstruction. It emphasizes the need to consider this condition in cases of recurrent, unexplained obstruction in adolescents and young adults. A high index of clinical suspicion, supported by appropriate imaging, is essential for timely diagnosis and prevention of morbidity. Diagnostic laparoscopy should be preferred in stable patients, as it not only confirms the diagnosis but also enables definitive management through careful adhesiolysis and release of entrapped bowel loops. When performed with surgical expertise, a minimally invasive approach can result in complete recovery without complications. Early recognition and timely laparoscopic intervention significantly reduce morbidity and ensure excellent clinical outcomes.
